# The UK Quality and Outcomes Framework pay-for-performance scheme and spirometry: rewarding quality or just quantity? A cross-sectional study in Rotherham, UK

**DOI:** 10.1186/1472-6963-9-108

**Published:** 2009-06-28

**Authors:** Mark Strong, Gail South, Robin Carlisle

**Affiliations:** 1 School of Health and Related Research, University of Sheffield, Regent Court, Sheffield, S1 4DA, UK; 2 Breathing Space Primary Care Respiratory Centre, Badsley Moor Lane, Rotherham, S65 2QL, UK; 3 Rotherham Primary Care Trust, Oak House, Moorhead Way, Rotherham, S66 1YY, UK

## Abstract

**Background:**

Accurate spirometry is important in the management of COPD. The UK Quality and Outcomes Framework pay-for-performance scheme for general practitioners includes spirometry related indicators within its COPD domain. It is not known whether high achievement against QOF spirometry indicators is associated with spirometry to BTS standards.

**Methods:**

Data were obtained from the records of 3,217 patients randomly sampled from 5,649 patients with COPD in 38 general practices in Rotherham, UK. Severity of airflow obstruction was categorised by FEV1 (% predicted) according to NICE guidelines. This was compared with clinician recorded COPD severity. The proportion of patients whose spirometry met BTS standards was calculated in each practice using a random sub-sample of 761 patients. The Spearman rank correlation between practice level QOF spirometry achievement and performance against BTS spirometry standards was calculated.

**Results:**

Spirometry as assessed by clinical records was to BTS standards in 31% of cases (range at practice level 0% to 74%). The categorisation of airflow obstruction according to the most recent spirometry results did not agree well with the clinical categorisation of COPD recorded in the notes (Cohen's kappa = 0.34, 0.30 – 0.38). 12% of patients on COPD registers had FEV1 (% predicted) results recorded that did not support the diagnosis of COPD. There was no association between quality, as measured by adherence to BTS spirometry standards, and either QOF COPD9 achievement (Spearman's rho = -0.11), or QOF COPD10 achievement (rho = 0.01).

**Conclusion:**

The UK Quality and Outcomes Framework currently assesses the quantity, but not the quality of spirometry.

## Background

Good quality management of chronic obstructive pulmonary disease (COPD) requires the use of spirometry for diagnosis, staging and ongoing monitoring[1]. Spirometry can be undertaken successfully in primary care if staff are appropriately trained[2]. However, poorly performed spirometry has the potential to cause misdiagnosis or the misclassification of the severity of airflow obstruction, and lead to inappropriate therapy and unnecessary patient anxiety[3].

Chronic obstructive pulmonary disease is included as a clinical domain within the pay-for-performance UK general practitioner "Quality and Outcomes Framework" (QOF) contract, first introduced in 2004. The explicit aim of QOF is to "reward contractors for good practice through participation in an annual quality improvement cycle"[4]. During the period of our study practices were paid through the contract for holding a COPD register, confirming the diagnosis of COPD with spirometry, recording FEV1, checking inhaler technique and offering flu vaccination.

GP practices have achieved highly on QOF COPD criteria. Across England 96.0% of available COPD points were obtained in the financial year 2006–7, worth approximately £33.5 million in incentive payments[5]. In Rotherham, the achievement was 94.5% of available points, and resulted in the allocation of approximately £220,000 of local NHS resources. This high level of achievement could be seen as an indication that the quality of care for people with COPD in English general practice is high. However, this conclusion can only be drawn if QOF does indeed measure "quality". A study conducted in Wales in the year preceding the introduction of the contract found that in approximately two fifths of the practices with spirometers, staff were not confident in their use[6], suggesting that universally high quality spirometry may have been difficult to achieve in the first years of the contract. This study assesses the quality of spirometry in primary care in Rotherham against evidence based standards published in national guidance documents, and measures the association between this measure of quality and QOF achievement at a practice level.

## Methods

### Sample

We obtained a register of all patients with a coded diagnosis of COPD from each of the 39 general practices in Rotherham Primary Care Trust (PCT). One small specialist practice that provides care to Rotherham's asylum seeker and homeless population had no patients coded as having COPD and was therefore removed from the study. In the remaining 38 practices we created two levels of sample. Firstly, a larger sample was used to record the proportions of patients categorised as having mild, moderate or severe COPD, the proportions of patients who had undergone spirometry, and the proportions of patients categorised as having mild, moderate and severe airflow obstruction. Secondly, a smaller sub-sample of notes was examined in order to assess the quality of spirometry.

The first-level sample was constructed as follows. In an initial pilot practice we randomly selected approximately 50% (161) of the patients with COPD, followed by two further pilot practices in which 90% (140 and 125) of patients were randomly selected. Thereafter, in the main study, we took random samples of 100 patients with COPD from each practice (unless there were fewer than 100 patients with COPD, in which case all patients on the practice COPD register were included). The smaller second-level sub-sample of patients contained a randomly selected subset of one in five patients from the first-level sample, stratified by practice, with a minimum of twenty patients from any single practice. For both levels of the sample, patients were selected using computer generated random numbers.

### Data collection

Data were obtained in the period between October 2006 and February 2007 by a small team of specialist nurses who visited each practice and searched paper records by hand, and computer records electronically.

### Analysis

From the larger first-level sample of notes we determined the proportion of patients with COPD who had had spirometry (ever, and in the previous 12 months), and the proportion of patients who had had their airflow obstruction categorised as mild, moderate or severe. We also used the most recent FEV1 results in the notes to categorise patients according to NICE criteria for categorising airflow obstruction (FEV1 50–80% of predicted, mild obstruction; FEV1 30–49% predicted, moderate obstruction; FEV1 < 30% predicted, severe obstruction)[1]. We compared categorisation on this basis with the clinical categorisation recorded in the notes. Cohen's Kappa statistic was used to assess agreement.

From the one in five randomly selected sub-sample of notes we determined the proportion of patients in whose notes there was evidence that spirometry had been carried out to British Thoracic Society (BTS) standards (three consistent readings of which two were within 5% or 100 mls)[7]. We excluded cases where the most recent spirometry had been performed in secondary care, and where the trace had faded such that the quality of the spirometry was indeterminate.

We recorded practice level achievement against the COPD QOF indicators (COPD 1, 8, 9, 10 and 11) for the 38 practices in 2006–07 (see table 1). We examined for an association between the quality of spirometry as measured by adherence to BTS guidelines for spirometry and QOF achievement against the two spirometry related indicators, COPD 9 and COPD 10, using Spearman's correlation coefficient.

**Table 1:** QOF COPD indicators (2006–7)

**Indicator**	**Details**	**Points available**
COPD 1	The practice can produce a register of patients with COPD	3
COPD 8	The percentage of patients with COPD who have had influenza immunisation in the preceding 1 September to 31 March	6
COPD 9	The percentage of all patients with COPD in whom diagnosis has been confirmed by spirometry including reversibility testing	10
COPD 10	The percentage of patients with COPD with a record of FeV1 in the previous 15 months	7
COPD 11	The percentage of patients with COPD receiving inhaled treatment in whom there is a record that inhaler technique has been checked in the previous 15 months	7

Notes: indicators numbered COPD 2–7 were included in previous years' contracts and are now redundant. Points are worth approximately £124 for an average practice.

We adjusted the PCT level proportions to allow for the practice level stratified sampling method[8], and confidence intervals for proportions were calculated using the Normal approximation. Bracketed intervals following point estimates are 95% confidence intervals unless stated otherwise. All analyses were carried out in R 2.9.0[9].

### Ethics approval

Ethics approval for this study was provided by Rotherham NHS Research Ethics Committee (ref number 06/Q230636).

## Results

At the time of the study practice list sizes ranged from 1,323 to 20,668. The crude prevalence of COPD ranged from 1.0% to 4.0% of the practice population, and the number of people on the QOF COPD register at each practice ranged from 22 to 395. Across the PCT the total number of COPD patients was 5,649, representing a mean PCT prevalence of 2.2%. This is approximately 50% higher than the crude prevalence in England of 1.4%[5].

3,217 (57%) sets of notes were selected for the first-level sample. The mean age of those sampled was 69 years (sd = 11 years) for both males and females. The current smoking prevalence was 31% (95% CI 29%–34%) in men, and 39% (95% CI 37%–41%) in women.

In 81% (80%–82%) of cases we found that spirometry had been performed at some point in the past, and in 50% (49%–51%) this was within the previous 12 months. Disease severity had been categorised by a clinician in 53% (51%–54%) of notes. Of those categorised, 45% (42%–48%) were categorised as mild, 35% (32%–38%) as moderate and 19% (16%–21%) as severe.

In 42% (40%–43%) of cases both a clinician categorisation of COPD and a spirometry result were recorded in the notes. When we categorised airflow obstruction according to the most recent FEV1 (% predicted) result, we found that patients tended to be categorised clinically as having either the same or more severe disease than was suggested by their most recent spirometry result (table 2). Agreement between clinician recorded disease severity and airway obstruction according to the spirometry results was low (Cohen's kappa = 0.34, 0.30 – 0.38). Approximately 12% of patients had spirometry results that did not appear to be consistent with a diagnosis of COPD.

**Table 2:** Agreement between COPD categorisation by spirometry, and COPD categorisation by clinician

	**COPD category by FEV1 (% predicted) result**				
**Clinician categorisation of COPD**	Normal	Mild	Moderate	Severe	Total

Normal	0	0	0	0	0
Mild	140	470	50	13	673
Moderate	17	218	235	13	483
Severe	5	23	105	97	230

Total	162	711	390	123	1386

A random sample of 761 (13.5%) sets of notes, stratified by practice, was selected for more detailed analysis. The most recent spirometry had been conducted in secondary care in 38 (5%) cases. Where spirometry had been performed in the practice, BTS standards (three readings, two of which were within 5% or 100 mls) were met in 31% (27%–35%) of cases. We excluded four (0.5%) cases where the spirometry trace had faded or was otherwise unclear. Adherence to BTS standards ranged from 74% of cases in one practice, to 0% in seven practices.

Overall, the 38 Rotherham practices achieved 94.5% (range 42.7% to 100%) of the QOF points available in the COPD domain in 2006–7, slightly lower than the national average achievement of 96.0% (table 3). At a practice level there was no correlation between the quality of spirometry as measured by adherence to the BTS standard (three readings, of which two were within 5% or 100 ml) and QOF achievement for spirometry as measured by either QOF indicator COPD 9 (Spearman's correlation coefficient = -0.11, *p *= 0.51), or indicator COPD 10 (Spearman's correlation coefficient = 0.01, *p *= 0.94).

**Table 3:** Overall QOF achievement against COPD indicators for Rotherham and England.

**Indicator**	**Mean achievement, points per practice, as percentage of points available**	
	**Rotherham (practice range in brackets)**	**England**

COPD 1	100%	99.7%
COPD 8	99.1% (75.7% to 100%)	98.4%
COPD 9	97.4% (28.4% to 100%)	97.4%
COPD 10	89.5% (16.7% to 100%)	93.0%
COPD 11	89.0% (43.3% to 100%)	93.5%
		
**Overall**	**94.5% (42.7% to 100%)**	**96.0%**

## Discussion

### Main findings

In 2006–7, practices in Rotherham achieved highly against the two QOF indicators that relate to spirometry, with the COPD 9 criteria met for 97.4% of the patients on COPD registers, and the COPD 10 criteria met for 89.5% of patients. However, in only 31% (95% CI 27%–35%) of cases were we able to find evidence of spirometry to BTS standards, and 12% of patients on COPD registers had FEV1 (% predicted) results recorded that did not support the diagnosis of COPD. There was no correlation at practice level between the QOF achievement against the two spirometry related indicators, and the quality of that spirometry. When we compared clinical categorisation of COPD severity with airflow obstruction severity we found that patients tended to be categorised clinically as having either the same or a higher severity of COPD than their category of airways obstruction severity.

### Limitations

Our assessment of quality of care was based on information that we were able to extract from the paper and electronic records held by the practices. Incompleteness or inaccuracy in the recording of care by a practice would limit the validity of our assessment of quality. It is possible that records were complete and accurate, but that we were unable to extract the information we needed, due, for example to problems in clinical coding. However, we would argue that correct coding to allow the easy extraction of important clinical data is itself good practice, and related to quality of care.

We found that in 50% (49%–51%) of patients we could find evidence of spirometry within the previous 12 months. This is rather lower than the reported achievement of 89.5% against the QOF indicator COPD 10, which records the proportion of patients in whom there is a record of FEV1 in the previous 15 months. This could have been due to the difference in timescales, but also may be related to the fact that QOF achievement is based only on the presence of a clinical code attached to the patient's electronic record, whereas we required evidence either from a hospital letter that spirometry had been performed, or the presence of the trace or readings.

Our study sample is representative of the population of people with COPD in only one health district, and the findings may not therefore be generalisable to the whole of the UK. It is unlikely, however, that these findings apply uniquely to Rotherham, an area where COPD care is high on the agenda of the local health community[10].

There is no single measure of COPD severity, and a patient's experience of the disease can be heavily influenced by, for example, the frequency of exacerbations, exercise tolerance, presence of co-morbidities and a range of psychological factors. Two people with the same degree of airflow obstruction may therefore experience very different levels of disease severity. It is not surprising then that we found a difference between clinical categorisation and airflow severity categorisation, and this confirms that airflow obstruction severity alone is not necessarily an adequate measure of COPD severity. However, spirometry plays a central role in the diagnosis and management of COPD and is recommended by NICE[1]. We feel therefore that this is strong justification for supporting primary care clinicians to conduct spirometry to nationally agreed standards.

### Previous studies

The relationship between QOF and spirometry has not been evaluated previously, though there are studies that have examined the relationship between QOF and other aspects of quality of care. A small study in two general practices in South East England found no relationship between QOF achievement and adherence to guidelines for people with stroke[11]. Poor data quality was cited as the probable reason for the apparent lack of quality of care. A larger study across two PCTs found that the associations between QOF and health outcomes (hospital admissions for a range of conditions and all-cause mortality) were small and inconsistent[12]. This finding may not be surprising given the relatively weak association between quality of care and outcomes, even after risk adjustment, due to the many other factors that influence outcomes[13].

QOF has been a step forward towards the systematic population wide management of COPD because, for the first time, practices hold registers of all their patients diagnosed with COPD. However, this study has highlighted a number of worrying results. The QOF contract represents a substantial investment in UK general practice and has caused significant shifts in priorities in primary care[14]. The recording of spirometry data in general practice increased markedly after the introduction of the 2003 QOF contract and 2004 NICE guidance (from 18% to 62% according to one large study)[15]. However, the value of this increase in recording to patients will only be maximised if the spirometry itself is carried to high quality standards.

## Conclusion

Our results suggest that the current Quality and Outcomes Framework contract measures the quantity, but not necessarily the quality of spirometry, and as such represents a lost opportunity to further improve COPD care. Possible solutions could be to revise the QOF criteria to include more explicit quality linked indicators, or alternatively, to give a much greater emphasis to alternative mechanisms of quality improvement such as clinical audit.

## Competing interests

The authors declare that they have no competing interests.

## Authors' contributions

RC and GS conceived the idea for the study. RC wrote the study protocol and gained ethical approval. GS led the data collection. MS analysed the data and drafted the manuscript. All three authors contributed to revisions and have approved the final draft.

## Pre-publication history

The pre-publication history for this paper can be accessed here:


